# Backward Displacement of the Womb and the Mineral Salts of Our Foods

**Published:** 1913-08-09

**Authors:** James Oliver

**Affiliations:** Gynæcologist to the Hospital for Women and Consulting Gynæcologist to the Ilford Emergency Hospital.


### BACKWARD DISPLACEMENT OF THE WOMB AND THE MINERAL SALTS OF OUR FOODS.

By JAMES OLIVER, M.D., F.R.S. (Edin.), F.L.S., Gynæcologist to the Hospital for Women and Consulting Gynæcologist to the Ilford Emergency Hospital.

Admittedly it is impossible to estimate precisely and define accurately the natural inclination of the uterus, but for all practical purposes we may accept the following description of the normal position of this organ, as given in Quain's Anatomy. "Its upper end is directed upwards and forwards, the lower downwards and backwards; so that its axis corresponds with that of the inlet of the pelvis and forms an angle or sudden curve with the axis of the vagina, which corresponds more nearly with that of the outlet of the cavity." It is very evident and will readily be conceded that the maintenance of this attitude depends upon tissue turgor and tissue strain, and that the elasticity of the walls of the various cellular elements concerned must play an important part in the phenomenon. Whatever, therefore, impairs the elasticity or lessens the turgescence of those tissues will tend to cause the organ to droop, and it is then extremely liable to become displaced backwards. Everyone is much more familiar with the occurrence of phenomena of this kind in plants than in animals, for daily it is remarked that flowers droop when the turgidity of the stalks can no longer be duly maintained, owing to the loss of water. Thus it happens, too, that after death the uterus is so very commonly found overlying Douglas's pouch, with its fundus in contact with the rectum. On the other hand, again, it is well known that the retroverted gravid uterus will in the majority of cases spontaneously assume and maintain the erect attitude after about the eighth or tenth week of pregnancy, and it is quite possible that this alteration in the position of the organ is due, not to any shortening or any greater efficacy *per se* of the round ligaments, but is caused by increased turgor and increased tissue tension brought about by the activation of the oosperm.

The enormous increase in the bulk and weight of the uterus which takes place in consequence of an actively progressing utero-gestation is entirely attributable to osmosis, and is very largely due to an increase in the liquid content of the organ. Now in both the vegetable and the animal world tissue tensions are dependent very especially upon osmotic phenomena, and in the case of our own bodies it is the mineral substances which play the all-important *rôle* in maintaining a normal composition and osmotic pressure in our liquids and tissues.

In consequence, moreover, of untoward innovations during recent years, the mineral content of many of our more important foodstuffs is now so lowered or so chemically altered that a form of mineral hunger is springing up which is exerting a most prejudicial influence upon our nervous constitution and upon such an organ as the uterus, seeing that the maintenance of the normal position of this organ depends so largely upon the adequacy and efficiency of the available mineral salts entering our bodies. The rational treatment of backward displacement of the womb aims, therefore, at combating this new form of mineral hunger, which is fraught with such danger to our bodies.

